# Intrinsically motivated humans and agents in open-world exploration

**DOI:** 10.1073/pnas.2612366123

**Published:** 2026-11-04

**Authors:** Aly Lidayan, Yuqing Du, Eliza Kosoy, Maria Rufova, Pieter Abbeel, Alison Gopnik

**Affiliations:** ^a^Berkeley AI Research Lab, Computer Science Department, University of California Berkeley, Berkeley, CA 94706; ^b^Psychology Department, University of California Berkeley, Berkeley, CA 94706

**Keywords:** exploration, empowerment, children, AI agents

## Abstract

What drives exploration? Understanding the intrinsic motivation to explore is a long-standing challenge in both cognitive science and AI. In this study, we directly compare adults, children aged 8 to 12 y, and AI agents exploring a complex, open-ended environment called Crafter (1). We study how three common information-theoretic intrinsic objectives—entropy, information gain, and empowerment—relate to their behavior. We find that all three are positively correlated with human exploration progress for both children and adults, although entropy and empowerment are more strongly related to exploration progress in children than information gain. Furthermore, across agents and humans we observe that entropy initially increases rapidly, then plateaus, while empowerment increases continuously, suggesting that in early exploration they look for novel states, while in advanced exploration they look for states they can control. Finally, we find preliminary evidence that private speech about goals may aid exploration in children. Our data are available at https://github.com/alyd/humans_in_crafter_data.

## Significance

How and why do we spontaneously explore the environment—even without any external rewards? What could lead artificial agents to spontaneously explore in the way that humans do? One idea is that people seek intrinsic rewards such as entropy, information, or empowerment, and this drives exploration. Drawing from both developmental psychology and AI, we compare exploratory behavior in children, adults, and AI agents navigating the same open-ended virtual world. Our findings contribute to theoretical debates about the nature of exploration and have practical implications for the development of AI agents.

Human beings explore new environments remarkably effectively, even without external rewards (2). Even young children explore spontaneously and intelligently (3, 4). This suggests that exploration may be a foundational human capacity that does not require explicit instruction or training. Exploration without external rewards is also crucial for artificial agents in unstructured, open-ended, or nonstationary environments where there is little direct feedback. How to give artificial agents exploration abilities is still an open question (5). One approach is to train agents with reinforcement learning (RL) to maximize intrinsic epistemic rewards, which incentivize visiting promising states or gaining valuable experiences (6), often taking inspiration from information theory and human curiosity (7, 8).

However, we still do not know how intrinsic rewards might actually motivate human exploration, and how that exploration compares to that of RL agents in open-world settings (9, 10). This is partly because open-ended exploration, by its very nature, is difficult to quantify. If we simply watched children’s spontaneous exploration in a playroom, for example, it might be hard to systematically and quantitatively analyze their behavior in computational ways. In contrast, the participants in our studies produced an average of over a thousand actions each but the virtual environment allowed us to automatically characterize and analyze those actions quantitatively. Similarly, it would be difficult to introduce AI agents with the same affordances as human children into a physical environment, but we can introduce them to exactly the same virtual environment as the children.

In the current study, we collect spontaneous exploratory play data from adults, 8 to 12 y old children, and RL agents in the same open-ended virtual environment—Crafter (1). Crafter is a “sandbox game” based on the popular game Minecraft. Unlike a typical video game, there is no obvious objective or score. Instead, the environment presents a wide breadth of activities to explore. Players move through a 2D grid (Fig. 1, *Left*), foraging for food and water, collecting materials, crafting tools, and defending against monsters, without knowing any of the mechanics in advance. Some activities are prerequisites for others, so that players can explore in depth as well as breadth (Fig. 2). For example, a player may collect wood, from which they may craft a wooden pickaxe, which can then be used to mine for coal. We can automatically record precisely which actions the human participants and AI agents take, and which states they reach, so we can compare their behavior directly.

**Fig. 1.**
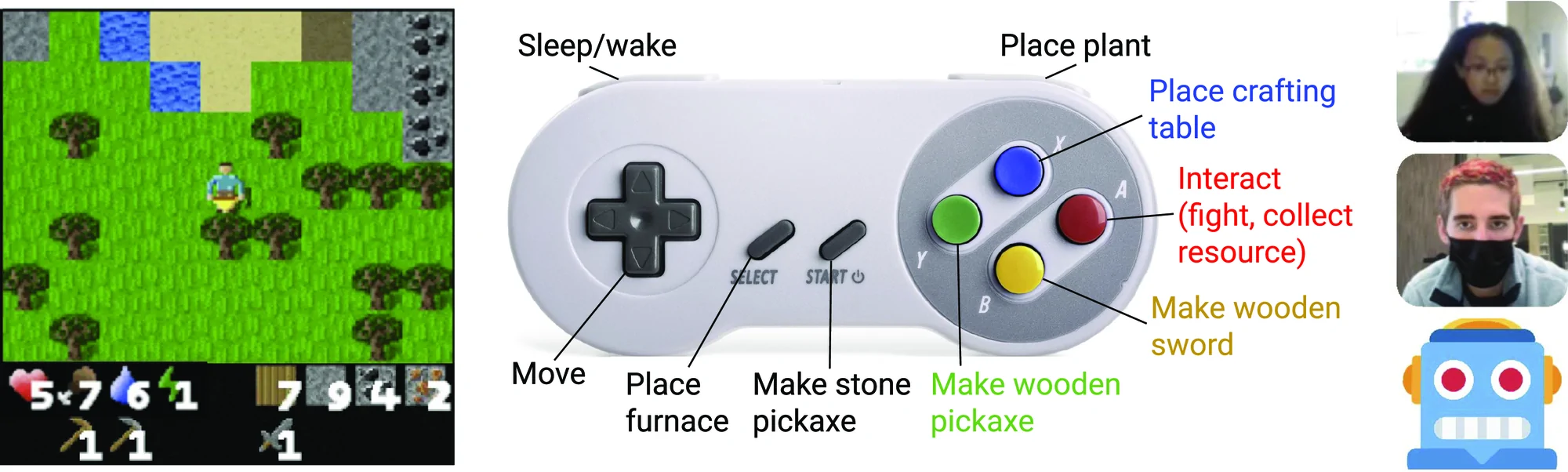
*Left*: Example screen from Crafter [1]. The player is at the center of the viewing window; the yellow arrow shows which direction they are facing. The black panel below displays the player’s status and inventory. In the top row, their health, food, water, and energy status are on the left and the raw materials they have collected are on the right. The tools built so far are in the bottom row. *Center*: Actions available to the human participants and RL agents. *Right*: We compare behaviors of children, adults, and RL agents.

**Fig. 2.**
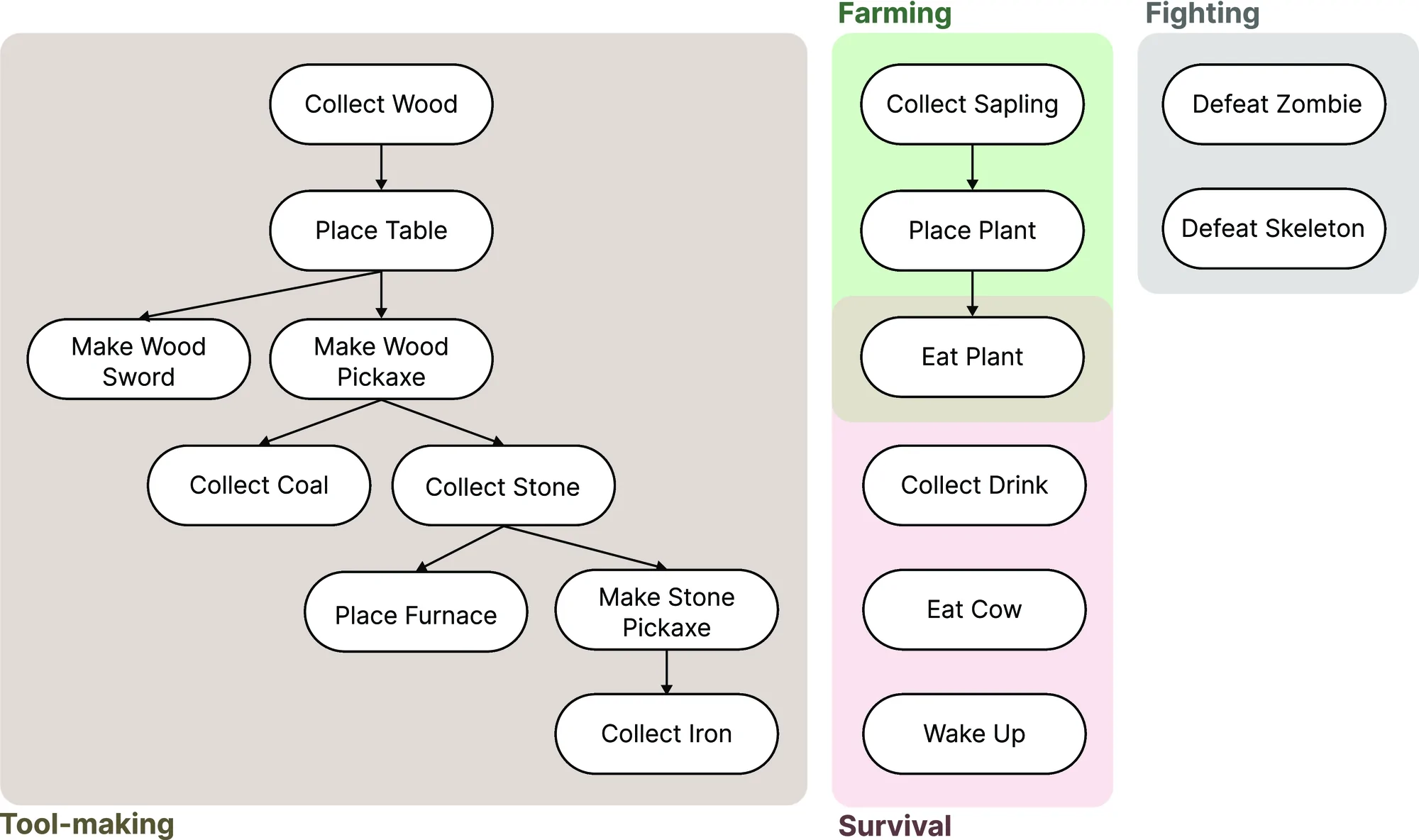
Achievements that can be reached in our version of Crafter, roughly clustered into four main categories. Arrows show dependency relations. This information is not visible to the human participants or RL agents.

We analyze how well the exploration progress of humans and RL agents correlates with three popular information-theoretic intrinsic objectives: *entropy*, *information gain*, and *empowerment*. Entropy measures the breadth of the distribution of states of the world that an agent visits (11). Information gain measures the amount of information an agent gathers through different behaviors, as a function of how many times they have been attempted in the past (12). Empowerment maximizes the mutual information between actions and outcomes (13). So to maximize empowerment an agent needs to vary their actions in a way that leads to parallel variation in outcomes. As a result, empowerment measures how much the agent can influence and control the environment. We also explore how behavior changes over time as participants play.

We also transcribe human utterances during play, and find significant positive correlations between children’s performance and their frequency of private speech utterances, particularly those verbalizing goals. This supports prior work in psychology which has suggested that private speech could play an important role in children’s creative problem-solving (14–18) and that proposing goals helps provide structure for open-ended exploration and play (19–21).

## 1.1. Exploration in AI Agents.

Approaches to exploration in reinforcement learning range from occasionally taking random actions (e.g., epsilon-greedy exploration) to automatic curriculum learning (22) and goal-setting (23–26). Adding intrinsic rewards is a particularly versatile method for motivating RL agents to explore promising states or gather valuable experiences (6). These rewards are often based on information-theoretic measures such as entropy, information gain, and empowerment, which can be applied to a wide variety of domains (7). Entropy rewards incentivize agents to explore a diverse spread of states or actions (27–29). Information gain rewards encourage gathering information about the environment and its dynamics (12), e.g., by acting in unfamiliar states that the agent cannot yet predict well (8, 30–32). Empowerment is maximized when the agent can choose from many distinct actions, each with a predictable consequence (13, 33), encouraging exploration that increases the agent’s influence over the environment (34, 35).

Intuitively, in our Crafter game an agent might maximize entropy by putting itself in as many different configurations (different combinations of location, health status, and inventory contents) as possible. It might maximize information gain by having maximally informative experiences, such as taking the least frequently tried actions from the least frequently visited configurations, and it might maximize empowerment by putting itself in configurations from which it has the most choice and control over future configurations.

However, even with sophisticated intrinsic objectives, RL agents generally lag far behind human exploration efficiency (2, 36). In some settings, intrinsic rewards can even harm exploration by unintentionally incentivizing degenerate behaviors, such as fixation on sources of random noise in the environment (37).

## 1.2. Exploration in Humans.

Developmental researchers have shown that even young children spontaneously explore in systematic and rational ways (38–40); see Schulz (41) for a review. In addition, prior work suggests that children may be especially exploratory, sometimes more exploratory than adults (4, 42). As in the AI literature, a variety of intrinsic objectives have been proposed to underlie human exploration (9, 10). Adults and children are attracted to novelty (43–46), which is related to increasing the entropy of the distribution of visited states. There is also evidence that both adults and children seek experiences that result in gaining more information about the environment (47–49). Brändle et al. (50) found that adults tried to maximize empowerment in an alchemy game, creating elements that increased the number of available reactions, and Melnikoff et al. (51) also found that adults maximized empowerment and that empowerment was associated with distinctive behaviors and feelings, particularly the feeling of unproblematic, spontaneous mastery that has been called “flow.” Yiu et al. (52) found that children and adults used empowerment as a cue for discovering causal structure.

Computational studies of exploration in cognitive science thus far have generally been limited to simple and unrealistic environments, typically with only a few different states and actions to choose from (53–55). Some more realistic studies have used mazes (56) or provided a larger, combinatorial state space (50). The Crafter environment offers a particularly rich and open-ended set of possibilities. Moreover, only a few studies have directly compared humans and agents (56–59), and only Kosoy et al. (56, 59) also included children. By comparing adults’, children’s, and AI agents’ exploration in this shared environment, we hope to illuminate whether intrinsic objectives such as entropy, information gain, and empowerment contribute to effective open-ended exploration.

## 2. Results

We conducted three main analyses on the recorded exploration data. First, we score how effectively adults, children, and each type of AI agent explored. Next, we analyzed to what extent human and agent behaviors increase entropy, information gain, and empowerment and how this relates to their exploration progress. In adults all three intrinsic objectives were correlated with exploration progress. For children, we found consistent significant correlations between exploration progress scores and both entropy and empowerment, but a weaker link to information gain. Last, we studied the relationship between private speech and exploration. In children, though not adults, we observed a strong positive correlation between the fraction of goal-based utterances and exploration proficiency.

### 2.1. Exploration Progress Scores.

We design five scores measuring different aspects of exploration (Table 1). Several are based on reaching semantically meaningful milestones called *achievements*; many achievements are prerequisites for others (Fig. 2), and all progress is reset when the player dies, at which point a new episode begins. Note that the achievements are not marked or rewarded in any external way, they are simply the outcomes of the agents’ spontaneous exploration.

**Table 1.** Description of exploration scores proposed for our study

Exploration Score	Definition
Mean Achievement Score	Average fraction of unique achievements reached per episode.
Map Coverage Score	Average fraction of the world map visited per episode.
Overall Achievement Score	Fraction of unique achievements unlocked over all episodes.
Overall Breadth Score	Fraction of steps taken in a breadth-first traversal of the achievement dependency tree (i.e., only counting achievements up to and including the first incomplete level of the tree).
Overall Depth Score	Normalized depth of the deepest achievement reached in the achievement tree.

Overall Achievement Score (AS) measures the fraction of all possible achievements reached at least once over all episodes. We break this down into Breadth and Depth to probe different possible exploration strategies. We also calculate the average fraction of possible achievements reached in each episode (Mean AS), which relates to how efficiently and consistently the player explores throughout the play session. Finally, we calculate the average fraction of grid cells visited in each episode’s procedurally generated world map as a measure of basic spatial exploration (Map Coverage).

### 2.2. Procedure.

Participants were allowed to play Crafter for up to 20 min. None of the participants had seen the game before. No game objective was given and no score was visible. Nevertheless, participants were highly engaged in the game; children took an average of over 1,300 actions and adults took over 2,700. For AI agent comparison, we trained RL agents with three different reward functions. We used two well-established intrinsic rewards from the RL literature: one based on entropy [*APT*, Liu and Abbeel (28)] and one on information gain [*NovelD*, Zhang et al. (32)]. In the extrinsic reward control (*Reward*), agents were specifically rewarded for reaching achievements in each episode. Successful RL training should lead the *Reward* agents to reach most achievements because they are directly incentivized to do so, so they serve to validate the training procedure. If an intrinsic reward is effective, in contrast, the corresponding agents should spontaneously explore many of the achievements as a side effect of maximizing the intrinsic reward.

Thirteen randomly initialized agents were trained per setting for 1 million time steps each, which is typical for RL training. We note that, although the humans had much less time to play Crafter, they benefit from prior life experience, whereas the agents were trained from scratch. Finally, we included agents that sampled actions uniformly at random (*Random*) as a baseline. See Section 4 for details.

### 2.3. Exploration Performance.

We present summary statistics across the human and agent data. Fig. 3 shows the distributions of exploration scores for each group. To compare the groups, we used Permutation tests on the means for the discrete Overall scores, and Wilcoxon Rank Sum tests for the continuous Mean AS and Map Coverage, and find the following significant differences (*P* < 0.05).

**Fig. 3.**
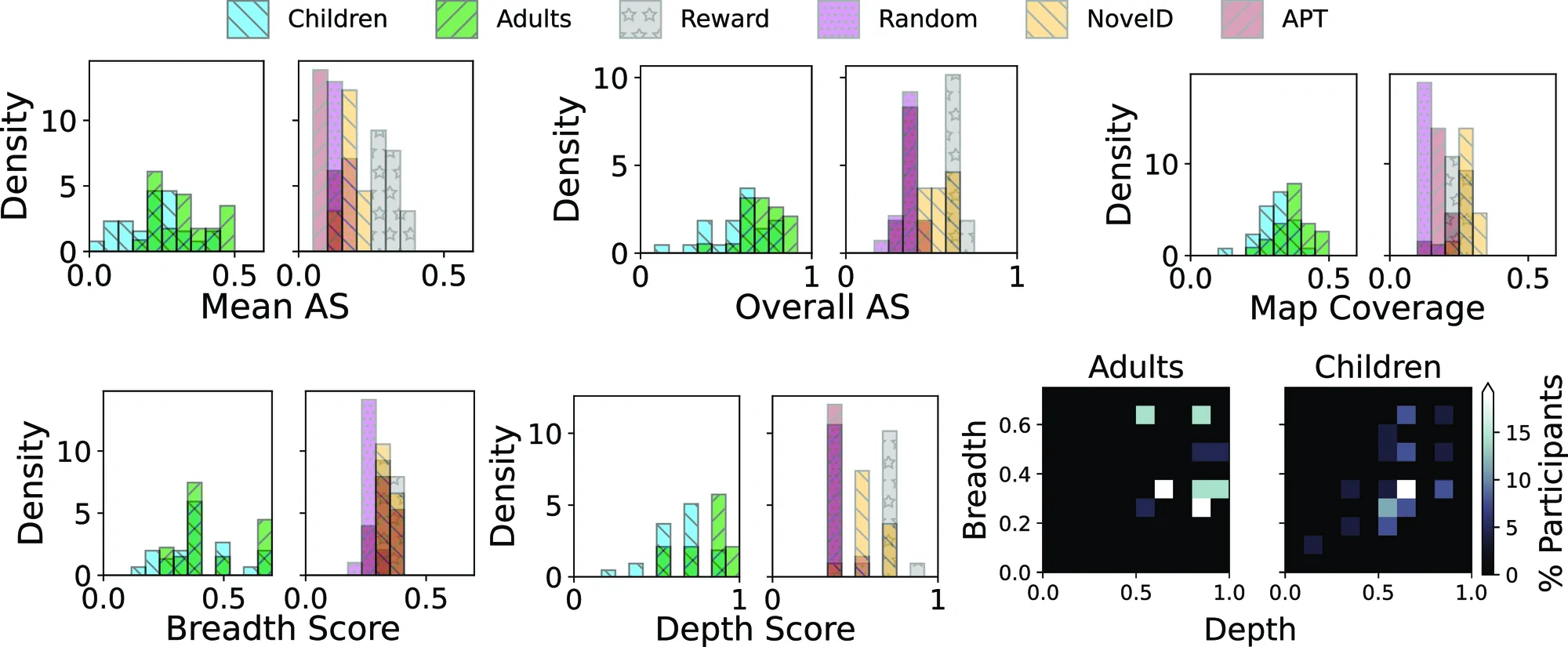
Summary density histograms for each exploration score. For clarity, the *Left* plot for each measure shows human scores and the *Right* plot shows agent scores.

The extrinsic *Reward* agents unsurprisingly outperformed the other agents on most scores, and scored higher than children and comparably to adults on Mean AS (the metric most similar to their extrinsic reward function). This indicates that our RL training procedure worked as intended. In addition, the *Random* agents performed worst across almost all metrics, showing that the intrinsic rewards were at least somewhat helpful for agent exploration. *NovelD* agents outperformed *APT* agents on all but Breadth, showing that its information gain-based reward was more effective for driving exploration than *APT*’s entropy-based reward. In fact, *NovelD* agents had comparable performance to the children, only performing worse on Map Coverage.

However, we found that adults explored the furthest in this challenging open-world environment, scoring higher than children on all but Breadth, and higher than the intrinsic agents across all scores. Children outperformed all agents on Map Coverage and outperformed *APT* on all scores but Breadth. The inferior performance of the intrinsic agents compared to both the extrinsic *Reward* agents and the intrinsically motivated adults suggests that humans’ open-ended exploration is driven by more effective forms of intrinsic motivation than those that the agents were trained with. In particular, as we will see below, human performance appeared to be driven by empowerment as well as the information gain and entropy that motivated the agents.

Analyzing the Breadth and Depth scores across humans, we found they were positively correlated for the children (ρ=0.51,P=0.01) but not the adults (ρ=-0.27,P=0.21). The 2D histograms in Fig. 3 show that the correlation is most apparent for the lower-scoring children, suggesting that it arises from a common cause of low effort or ability. Different exploration styles become apparent in more proficient players, some of whom prefer to build on existing progress while others make more diverse, independent discoveries.

### 2.4. Information-Theoretic Intrinsic Objectives.

We next characterized human and agent exploration with respect to the information-theoretic intrinsic objectives.

Entropy measures the diversity of all the states visited throughout gameplay. The state visitation distribution is defined by $p\left(s\right)=N_{s}/\sum_{s^{\prime }\in 𝒮}N_{s^{\prime }}$, where $N_{s}$ is the number of times the player was in state s. The entropy of this distribution is defined as

$$H\left(S\right)\;:=\;\overset{\;s\in 𝒮}{-\sum }p\left(s\right)\text{log}p\left(s\right).$$

The entropy at episode e is calculated over the visitation counts summed over all episodes up to and including e. The overall *Entropy* is reported over each player’s total experience, summing counts across all episodes.

Information Gain is a measure of how much information is gained on average over each episode, in terms of the novelty of the player’s behavior. The cumulative information gathered up to the end of episode e from taking action a from state s a total of $N_{s,a}^{e}$ times is measured as $log(1+N_{s,a}^{e})$. We use the log-count to approximate the diminishing novelty of repeated observations, as prior work has found it to perform similarly to more complex measures (2). The information gained just in episode e is the difference in total information between that episode and the previous episode, i.e., $log\left(1+N_{s,a}^{e}\right)-log\left(1+N_{s,a}^{e-1}\right)$. This is summed over all (s,a) pairs and divided by the total number of transitions occurring during that episode to get the average information gain per transition at episode e:

$$\begin{array}{c} IG^{e}\;:=\;\frac{\sum_{s,a}\text{log}\left(1+N_{s,a}^{e}\right)-\text{log}\left(1+N_{s,a}^{e-1}\right)}{\sum_{s,a}N_{s,a}^{e}-N_{s,a}^{e-1}}. \end{array}$$

The overall *Information Gain* is finally calculated as the mean of $IG^{e}$ over all episodes.

Empowerment can be interpreted as a measure of the amount of influence the agent has over the environment. The empowerment at state s is defined as the channel capacity between the agent’s actuators and the next state $s^{\prime }$ from s (60):

$$\begin{array}{c} E(s)\;:=\;\underset{p(a)}{\text{max}}I(S^{\prime };A|\;s)=\underset{p(a)}{\text{max}}\sum_{s^{\prime },a}\\ p(\;s^{\prime }|a,s)p(a)\text{log}\frac{p(s^{\prime }|a,\;s)}{\sum_{\tilde{a}\in \text{A}}p(s^{\prime }|\tilde{a},s)p(\tilde{a})}, \end{array}$$

where I is mutual information. In other words, the empowerment at a particular state is given by the maximum mutual information between actions a taken from that state and subsequent states $s^{\prime }$. The channel capacity is the maximum number of bits that can be communicated per action by the resulting change in game states. For each participant we compute transition probabilities $p(s^{\prime }|a,s)$ using observed transition counts $N_{s,a,s^{\prime }}$ accumulated across all episodes. Next state distributions from unexplored states and actions are treated as uniform (corresponding to low empowerment), thus this measure can be viewed as a player’s experienced empowerment. We only count single-step transitions $\left(s_{t},a_{t},s_{t+1}\right)$ because most meaningful transitions in Crafter occur over one time step. We use the Blahut-Arimoto (61) algorithm to approximate the channel capacity, as proposed in (33), and sum over all states to obtain the total *Empowerment* as $E=\sum_{s\in 𝒮}E\left(s\right)$.

When calculating all objective values, we used a reduced state representation to collapse the most semantically similar states, which is necessary to handle sparsity in our data. We combined the semantic label of the contents of the cell the player is currently facing, the contents of their inventory, and the increase in their status from the previous state, if any. This captures aspects of the environment that the player is most likely to be paying attention to and has direct control over, but avoids meaningless sources of variation in the state (e.g., slightly different positions of trees in the player’s surroundings). We used this representation to construct transition tables from the collected gameplay data, mapping each transition $\left(s,a,s^{\prime }\right)$ to the number of times it was experienced. We further filtered out movement transitions, focusing on direct attempts to influence the environment.

These measures are mathematically defined. But, intuitively, maximizing Entropy in the Crafter environment might include visiting as many states as possible, while maximizing Information Gain would involve trying as many state-action combinations as possible, and maximizing Empowerment would involve taking actions that led to more control of future states.

### 2.5. Achievement of Intrinsic Objectives and its Relation to Exploration.

We ran Wilcoxon Rank-Sum tests to compare the overall intrinsic objective values for each group of humans and agents. To assess the relationship between intrinsic objective values and exploration scores, we computed least-squares linear fits for each human group and used two-sided Wald tests to assess whether the slope differed from zero. The following differences and associations were significant at *P* < 0.05.

The humans attained higher levels of all three intrinsic objectives than *Random* agents. We did not include an empowerment-based agent, but it is notable that the human adults and children achieved high empowerment without any explicit incentive, reward, or training. The adults also surpassed all the trained agents on each intrinsic objective (Fig. 4). Children attained higher *Entropy* than *APT* and lower *Empowerment* than *NovelD*. On the remaining objectives they were comparable to the intrinsically motivated agents.

**Fig. 4.**
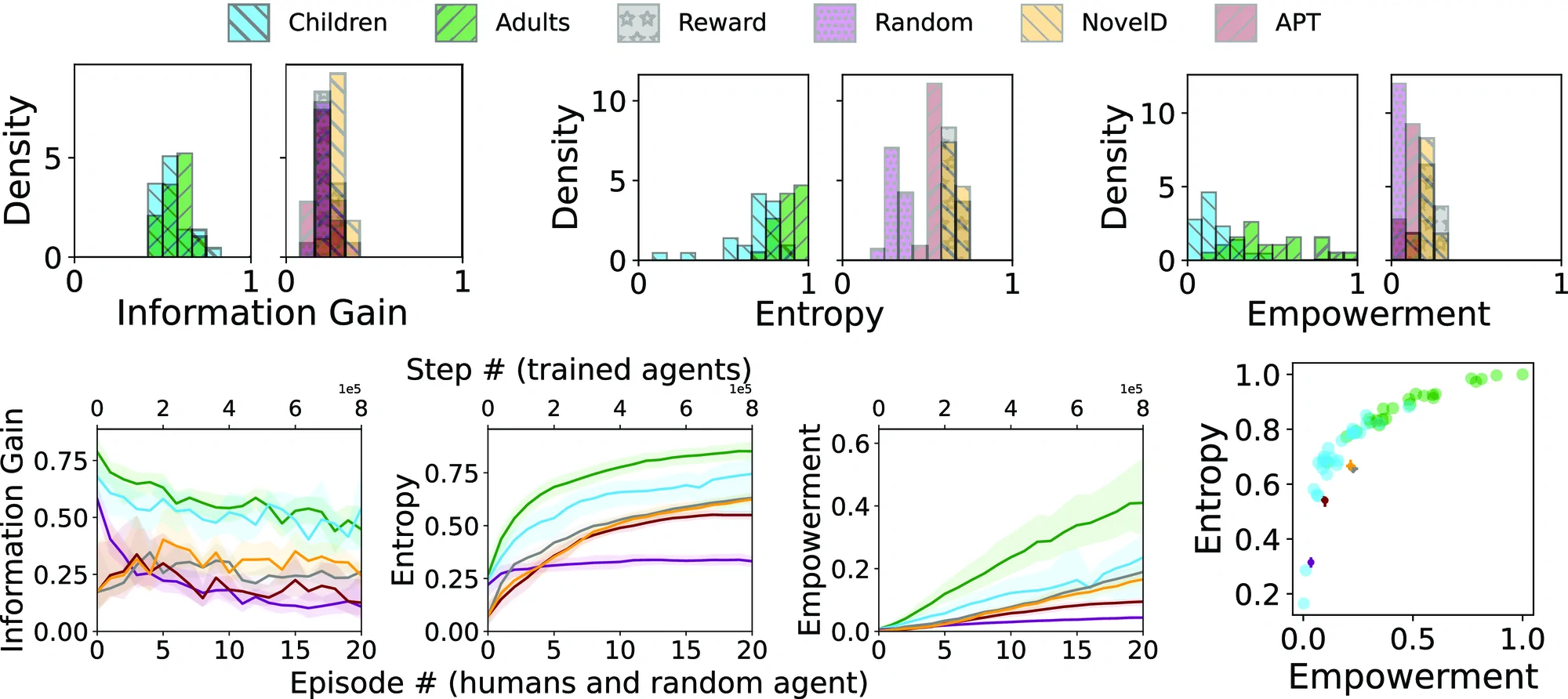
Histograms showing the distribution of each normalized information-theoretic objective attained by each group, their means over time, and a scatter plot of overall *Entropy* and *Empowerment* for each human participant and the mean for each agent type. Error bars and shaded regions indicate bootstrapped 95% CIs.

Furthermore, we found significant positive correlations between adults’ achievement-based exploration scores and each of those objectives (Fig. 5), suggesting that they may indeed help to motivate human open-ended exploration. Children’s overall achievement-based exploration scores were correlated with their Empowerment and Entropy but not with Information Gain (Fig. 5). This may be because it is possible to attain high *Information Gain* by trying many different actions at every state, even though most actions have no effect and thus do not lead to achievements. Children may have worse priors over which actions will be effective than adults, so that they try more actions while making less progress.

**Fig. 5.**
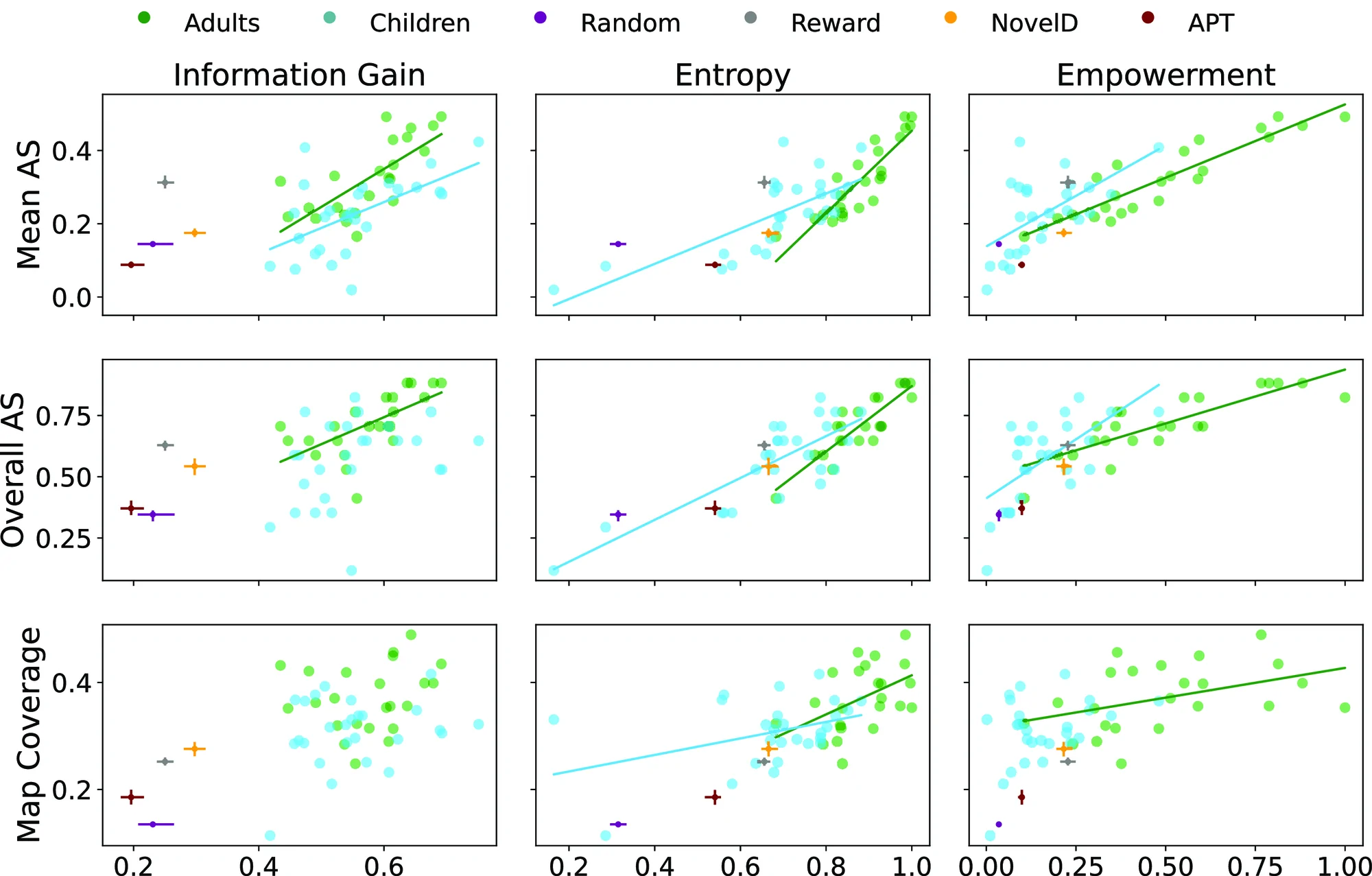
Information-theoretic objectives vs. exploration scores. We plot least-squares linear fits for the adults and children wherever the slope is significantly greater than zero (*P* < 0.05). Humans’ achievement-based exploration scores were significantly correlated with their *Entropy* and *Empowerment*. The mean and bootstrapped 95% CIs across random seeds are shown for each AI agent type.

Surprisingly, although *APT* agents were trained with an entropy-based reward, they performed worse than the other RL agents on all information-theoretic measures including *Entropy*. *NovelD* agents, which were trained with an information gain-inspired reward, surpassed *Reward* agents on *Information Gain* only. This may be because we calculate the measures over an abstract state space focusing on immediately controllable parts of the environment (Section 2.4), while *APT* and *NovelD* must work with the agent’s higher-dimensional raw inputs to estimate entropy and novel information (Section 4.4). This can motivate exploration of meaningless variation in environments as complex as Crafter, similar to the “Noisy TV” problem, where intrinsically motivated agents are transfixed by random images on a TV screen (37).

### 2.6. Temporal Dynamics of Intrinsic Objectives.

Plotting the objectives over time (Fig. 4, *Bottom Left*) shows that the *Information Gain* per episode predictably decreased for the humans as the most accessible observations get exhausted, but both adults and children maintained it at a significantly higher level than all the agents throughout their exploration. All groups increased their *Entropy* and *Empowerment* throughout exploration, though *Entropy* increased more rapidly early on before plateauing, whereas *Empowerment* increased at a steady rate. We also found that overall *Entropy* and *Empowerment* exhibit a nonlinear positive correlation, where *Empowerment* increases more steeply for players that attain high *Entropy* (Fig. 4, *Bottom Right*). These results suggest that *Entropy* could be a predominant objective earlier in exploration while there are still many novel states to observe, whereas *Empowerment* provides more signal once most states have been visited but there is still more to learn about how to predictably influence them.

### 2.7. Private Speech and Exploration.

We present a preliminary analysis of the relationship between private speech, exploration, and the intrinsic objectives (Fig. 6). We took transcriptions from each consenting participant and filtered for private speech utterances. Though many participants did not talk at all, a few uttered over 1,000 words of private speech. Children spoke to themselves almost twice as much as adults, with an average word rate of about 16 words/min compared to adults’ approximately 8 words/min, although both distributions had long tails (Fig. 6, *Left*).

**Fig. 6.**
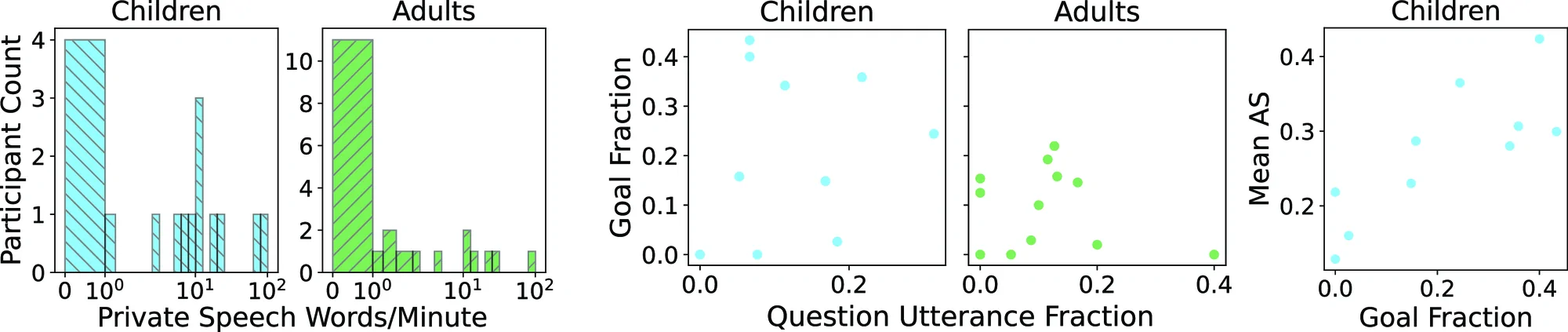
*Left*: the distribution of word rates from the subjects’ private speech utterances. *Center*: The fraction of private speech utterances that expressed questions vs. goals; children were on average more than twice as likely to express goals as adults. *Right*: The fraction of verbalized goals vs. Mean Achievement Score in children, which exhibited a strong correlation [*r*(8) = 0.8, *P* = 0.005, unadjusted].

We found weak positive associations between the children’s word rate and all intrinsic objectives and exploration scores, but they did not reach statistical significance. In addition, we note that a higher rate of private speech in children may simply reflect increased engagement with the game rather than a causal relationship.

Inspired by the rich literature on goal-directed exploration, we further investigated whether children who were more likely to verbalize goals during private speech explored more successfully. As a contrasting measure, we also analyzed how often children verbalized questions (see Table 2 for examples). Following prior work summarizing human data with LLMs (62), we used ChatGPT (gpt-3.5-turbo) to classify whether each utterance expressed a goal and/or question. To improve accuracy, we prompted for reasoning before making each classification. See *SI Appendix, Utterance Classification* for sample prompts and responses. To reduce the effects of noise, we limited our analysis to participants who made at least five utterances throughout their gameplay, leaving us with N=10 children and N=12 adults.

**Table 2.** Examples of private speech utterances

Utterance type	Examples
Questions	“How do I make a wooden pickaxe?,” “What does sleep mean?,” “What is that skeleton doing?,” “Can I go in water?,” “where is stone?”
Goals	“I need to mine some stuff,” “I wanna get the tree,” “I need meat!,” “gonna kill that stuff,” “now I can get some stones”

While the fraction of utterances containing questions was almost the same for children and adults at 12 to 13%, children were on average more than twice as likely to express goals as adults, at 21% vs. 10% (Fig. 6, *Center*). And while we found no significant correlation between the fraction of questions and any exploration scores, we found a striking positive correlation [r(8)=0.80,P=0.005, unadjusted] between the fraction of goal utterances and the children’s Mean Achievement Score (Fig. 6, *Right*). This suggests that explicit goal setting may indeed be important for children’s exploration, though we note that these analyses are only exploratory and should be confirmed in a larger sample. Moreover, the causal direction of the link is unclear—it is possible either that children who expressed more goals were therefore more effective explorers or that children who were more motivated to explore also were more likely to articulate goals.

## 3. Discussion

In this work we investigate the role that information-theoretic intrinsic motivation may play in driving open-ended exploration in complex environments. We find that measures of entropy and empowerment are consistently correlated with more prolific exploration in adults and children. However, agents trained on intrinsic rewards, even with many orders of magnitude more time with the environment, do not explore as far as adults. This suggests that intrinsically motivated agent exploration could be improved through better ways of approximating and incorporating entropy- and empowerment-based objectives. In particular, it might be helpful to design objectives that abstract away low-level variation to focus on more meaningful high-level structure. For example, joint embedding predictive architectures as advocated by Yann LeCun (63), may be used to learn to abstract away all but the features that are jointly the most predictive of and predictable in the future. We are also currently exploring how intrinsic motivation could lead to new representations, as well as leading to new actions and policies (64). In addition, we observe that *Entropy* increases rapidly before plateauing and *Empowerment* increases slowly and steadily throughout gameplay, which suggests that they may be even more effective when combined: *Entropy* could guide early exploration while *Empowerment* may provide more signal for advanced exploration.

We find that children underperform adults on most exploration metrics and intrinsic objectives except *Information Gain*. This suggests that children explore as many behaviors as adults but the things they try are less likely to work, possibly as a result of having worse priors about the possible dynamics of the environment. We also find preliminary evidence suggesting that children’s performance may be aided by overt verbalization: Children with higher word rates tend to perform better, and there is a positive correlation between the fraction of utterances specifically mentioning goals and the rate of reaching achievements. These results are consistent with prior findings in psychology that self-talk can help direct and focus problem solving in children, and findings in AI that agents that generate goals may explore more effectively (18, 24, 25). Comparing human exploration with that of autotelic AI agents is left for future work.

### 3.1. Limitations.

Although each participant in our study generated a large amount of structured data (an average of over 1,300 actions from each child and 2,700 from each adult), the relatively small number of participants limits broader conclusions about human exploration in different settings and populations. The sample size is even smaller for the private speech findings, which need to be confirmed with a larger sample in a preregistered study format. Moreover, the causal relation between more speech and exploration is not clear and needs to be tested further. Many of the children under 8 y old whom we tested had difficulty understanding the game, and thus more child-friendly environments would be helpful for studying exploration across development. Our calculations of the information-theoretic objectives are sensitive to the state representation used; although we tried to mimic human prior knowledge by using a relatively compact semantic representation, humans’ priors may allow them to compress the state space in even more effective ways.

### 3.2. Conclusions.

Most significantly, this work serves as a demonstration that it is possible to compare the spontaneous exploration of adults, children, and AI agents in the same complex, open-ended environment. Moreover, we show that formal information-theoretic measures such as entropy, information gain, and empowerment can help to explain behavior in all three groups and find intriguing preliminary evidence connecting children’s exploration with goal-directed speech. We hope that this work lays the groundwork for future work to collect larger datasets in even richer environments, and investigate the role of goals and abstractions in intrinsically motivated open-ended exploration.

## 4. Methods

### 4.1. Open-Ended Environment: Crafter.

We conduct our experiments in Crafter (1). Similar to Minecraft, Crafter allows the player to explore a wide range of activities. The player controls a character in a procedurally generated grid-world containing various resources that can be collected and used to replenish status levels (health, food, water, energy) or build tools (Fig. 1). Health decreases when the player is attacked by monsters, or any of the other status levels are at 0. The environment cycles between day and night; at night, the player’s view is darkened and more monsters are spawned. The episode ends when the health level reaches 0, resetting the player into a new randomly generated world with an empty inventory. All semantically meaningful changes in state that are under the player’s control are marked by *achievements*, some of which have multilevel prerequisites that must be satisfied within the same episode (Fig. 2). Several of the basic achievements, e.g., Collect Drink, are needed to stay alive, but the tool crafting achievements do not help with survival.

The available actions either move the player or interact with the environment. Interactions only affect the player’s status, inventory, and/or the cell that the player is directly facing, with the Interact action being the most versatile (used to eat, drink, cut trees, mine, or fight, depending on the contents of the cell). Seven additional actions each have unique effects, sometimes requiring prerequisites to be satisfied, e.g., Make Wood Sword converts wood in the player’s inventory into a wooden sword, and requires that they are facing a crafting table and have a sufficient amount of wood.

We modify the game to make it easier to master in a short play session and accessible via a conventional game controller. First, we slightly lengthen the fraction of an in-game day that is spent in daylight by changing the daylight function from $1-{\left|cos\left(\pi x\right)\right|}^{3}$ to $1-{\left|cos\left(\pi x\right)\right|}^{12}$. We also add an explicit “Game Over” screen when an episode ends to ensure participants are aware of when their character dies. Last, we slightly prune the action space, removing five of the most advanced actions from the original game as they were not feasible to map onto a conventional handheld game controller (Fig. 1, *Center*).

### 4.2. Participants.

In this preregistered, IRB-approved study (AsPredicted reference: 92521), we initially recruited 51 children aged 6 to 12 y from the Bay Area Discovery Museum (BADM), as well as 24 adults from the University of California, Berkeley campus aged 18 to 25 y (mean age: 24.8, female: 10, male: 14). Participants who did not complete at least one full episode of the game, or who received significant verbal interference from onlookers at the BADM, were excluded. The basics of the game, such as using the controller, turned out to be too challenging for younger children, so we only analyzed data from children who were at least 8 y old (N=26, mean age: 9.4, female: 11, male: 15). None of the participants had previously seen Crafter. 92% of children and 79% of adults had video game experience, with 77% of children and 54% of adults having played Minecraft at least once. We find that prior Minecraft experience did not have a large effect on the participants’ performance (*SI Appendix*, Fig. S1), as there are sufficient differences between the two games. There were also no age effects within the children. All research was conducted under University Of California at Berkeley Institutional Review Board Protocol # 2010-01-631, “Causal Learning in Children” and informed consent was obtained for all participants.

### 4.3. Human Data Collection Procedure.

Participants were first shown a short tutorial video on how the controller worked (Fig. 1, *Center*), without specifying any scoring system or objective. Participants were then allowed to play for as long as they wished, up to 20 min. All actions taken and the complete world state were recorded for every time step. Audio recordings from participants who consented to recording (17 children and 23 adults) were later transcribed manually with timestamps.

### 4.4. Agent Training Procedure.

We trained three types of RL agent and used one random agent as a baseline. At each time step, the random agent took no action with probability 0.475 to match the average action rate of the human players, and uniformly sampled all available actions otherwise. We trained two agents (*NovelD* and *APT*) with well-established intrinsic RL objectives. NovelD (32) incentivizes information gain by providing a large intrinsic reward at the boundary between familiar and novel regions, using Random Network Distillation (37) as a measure of state novelty. APT (28) uses a particle-based entropy estimator (65) to reward the agent for maximizing state entropy in a learned representation space. We did not train agents with empowerment-based intrinsic rewards due to the computational complexity of computing empowerment at scale. As a control, we also trained an extrinsic agent (*Reward*) with a hand-crafted reward function that explicitly rewarded it for unlocking in-game achievements (Fig. 2)—which are also the basis for most of our exploration performance metrics. This provided a reward of 1 every time an achievement was reached for the first time in each episode, alongside a small reward of ±0.1 every time the agent replenished or lost health.

Humans use their prior knowledge to process visual inputs into semantic representations—they identify one configuration of pixels as an axe and another as a tree—which aids performance [see Dubey et al. (57)]. To make the evaluation more comparable, the agent policy input was a simplified semantic representation of the game: discrete labels for the contents in each cell in the agent’s 9 × 8 viewing window, the status, and the inventory (Fig. 1, *Left*). All RL agents were trained with Rainbow DQN (66) for one million time steps, with epsilon-greedy exploration decaying epsilon from 1 to 0.01 on a linear schedule over 250,000 time steps. We trained each agent with 13 random initializations. Finally, for our behavioral analysis we subsampled 25 episodes evenly throughout the training trajectory of each agent to reduce the number of episodes to the same order of magnitude as the humans’ (which ranged from 5 to 28).

### 4.5. Declaration.

Ethics approval and consent to participate: All studies had full Institutional Review Board approval under the Gopnik Lab and required full consent to participate.

Consent for publication: All participants consented and all children’s names have been redacted from any data published. Some consented to partial or full data publication. The initial study was preregistered under AsPredicted reference: 92521.

## Supplementary Material

Appendix 01 (PDF)

## Data Availability

Our dataset of gameplay and transcripts, as well as analysis code are available at https://github.com/alyd/humans_in_crafter_data (67). We hope it will be a useful resource for future research.

## References

[1] D. Hafner, Benchmarking the spectrum of agent capabilities. arXiv [Preprint] (2021). 10.48550/arXiv.2109.06780 (Accessed 6 October 2026).

[2] B. Matusch, J. Ba, D. Hafner, Evaluating agents without rewards. arXiv [Preprint] (2020). 10.48550/arXiv.2012.11538 (Accessed 6 October 2026).

[3] A. P. Giron , Developmental changes in exploration resemble stochastic optimization. Nat. Hum. Behav. **7**, 1955–1967 (2023). 10.1038/s41562-023-01662-1 PMC10663152 37591981

[4] A. Gopnik, Childhood as a solution to explore–exploit tensions. Philos. Trans. R. Soc. Lond. B Biol. Sci. **2020**, 20190502 (1803). 10.1098/rstb.2019.0502 PMC7293160 32475327

[5] E. Hughes , Open-endedness is essential for artificial superhuman intelligence. arXiv [Preprint] (2024). 10.48550/arXiv.2406.04268 (Accessed 6 October 2026).

[6] A. Lidayan, M. D. Dennis, S. Russell, BAMDP shaping: A unified framework for intrinsic motivation and reward shaping. arXiv [Preprint] (2025). 10.48550/arXiv.2409.05358 (Accessed 6 October 2026).

[7] A. Aubret, L. Matignon, S. Hassas, An information-theoretic perspective on intrinsic motivation in reinforcement learning: A survey. Entropy **25**, 327 (2023). 10.3390/e25020327 PMC9954873 36832693

[8] J. Schmidhuber, A possibility for implementing curiosity and boredom in model-building neural controllers. Proc. Int. Conf. Simul. Adapt. Behav., 222–227 (1991).

[9] A. Ten, P. Y. Oudeyer, C. Moulin-Frier, Curiosity-Driven Exploration (The Science of Human Information Seeking, The Drive for Knowledge, 2022), p. 53.

[10] M. Pelz, C. Kidd, The elaboration of exploratory play. Philos. Trans. R. Soc. B **2020**, 20190503 (1803). 10.1098/rstb.2019.0503 PMC7293158 32475326

[11] F. Bourgault, A. A. Makarenko, S. B. Williams, B. Grocholsky, H. F. Durrant-Whyte, “Information based adaptive robotic exploration” in IEEE/RSJ International Conference on Intelligent Robots and Systems, (IEEE, 2002), vol. **1**, pp. 540–545.

[12] D. V. Lindley, On a measure of the information provided by an experiment. Ann. Math. Stat. **27**, 986–1005 (1956).

[13] A. S. Klyubin, D. Polani, C. L. Nehaniv, “Empowerment: A universal agent-centric measure of control” in IEEE Congress on Evolutionary Computation (IEEE, 2005), Vol. **1**, pp. 128–135.

[14] L. S. Vygotsky, Thought and Language (MIT press, 1962).

[15] L. Vygotsky, Thinking and Speech (Problems of General Psychology, The collected works of LS Vygotsky, 1987), p. 1.

[16] D. H. Schunk, Verbalization and children’s self-regulated learning. Contemp. Educ. Psychol. **11**, 347–369 (1986).

[17] G. Granato, A. M. Borghi, G. Baldassarre, A computational model of language functions in flexible goal-directed behaviour. Sci. Rep. **10**, 21623 (2020). 10.1038/s41598-020-78252-y PMC7729881 33303842

[18] S. W. F. Lee, Exploring seven- to eight-year-olds’ use of self-talk strategies. Early Child Dev. Care **181**, 847–856 (2011).

[19] J. Chu, L. E. Schulz, Play, curiosity, and cognition. Annu. Rev. Dev. Psychol. **2**, 317–343 (2020).

[20] G. Davidson, T. M. Gureckis, B. Lake, Creativity, compositionality, and common sense in human goal generation. Proc. Annu. Meet. Cognit. Sci. Soc. **44**, 3369–3375 (2022).

[21] G. Molinaro, A. G. Collins, A goal-centric outlook on learning. Trends Cogn. Sci. **27**, 1150–1164 (2023). 10.1016/j.tics.2023.08.011 37696690

[22] R. Portelas, C. Colas, L. Weng, K. Hofmann, P. Y. Oudeyer, Automatic curriculum learning for deep RL: A short survey. arXiv [Preprint] (2020). 10.48550/arXiv.2003.04664 (Accessed 6 October 2026).

[23] J. Wei , Chain-of-thought prompting elicits reasoning in large language models. Adv. Neural Inf. Process. Syst. **35**, 24824–24837 (2022).

[24] C. Colas, T. Karch, O. Sigaud, P. Y. Oudeyer, Autotelic agents with intrinsically motivated goal-conditioned reinforcement learning: A short survey. J. Artif. Intell. Res. **74**, 1159–1199 (2022).

[25] Y. Du , Guiding pre-training in reinforcement learning with large language models. arXiv [Preprint] (2023). 10.48550/arXiv.2302.06692 (Accessed 6 October 2026).

[26] J. Zhang, J. Lehman, K. Stanley, J. Clune, OMNI: Open-endedness via models of human notions of interestingness. arXiv [Preprint] (2023). 10.48550/arXiv.2306.01711 (Accessed 6 October 2026).

[27] E. Hazan, S. Kakade, K. Singh, A. Van Soest, “Provably efficient maximum entropy exploration” in International Conference on Machine Learning, (PMLR, 2019), pp. 2681–2691.

[28] H. Liu, P. Abbeel, Behavior from the void: Unsupervised active pre-training. Adv. Neural Inf. Process. Syst. **34**, 18459–18473 (2021).

[29] D. Yarats, R. Fergus, A. Lazaric, L. Pinto, “Reinforcement learning with prototypical representations” in International Conference on Machine Learning, (PMLR, 2021), pp. 11920–11931.

[30] R. Houthooft , Variational information maximizing exploration. Adv. Neural Inf. Process. Syst. **29**, 1109–1117 (2016).

[31] D. Pathak, P. Agrawal, A. A. Efros, T. Darrell, “Curiosity-driven exploration by self-supervised prediction” in International Conference on Machine Learning, (PMLR, 2017), pp. 2778–2787.

[32] T. Zhang , NovelD: A simple yet effective exploration criterion. Adv. Neural Inf. Process. Syst. **34**, 25217–25230 (2021).

[33] A. S. Klyubin, D. Polani, C. L. Nehaniv, “All else being equal be empowered” in European Conference on Artificial Life, (Springer, 2005), pp. 744–753.

[34] C. Salge, C. Glackin, D. Polani, Changing the environment based on empowerment as intrinsic motivation. Entropy **16**, 2789–2819 (2014).

[35] K. Gregor, D. J. Rezende, D. Wierstra, Variational intrinsic control. arXiv [Preprint] (2016). 10.48550/arXiv.1611.07507 (Accessed 6 October 2026).

[36] D. Hafner, J. Pasukonis, J. Ba, T. Lillicrap, Mastering diverse domains through world models. arXiv [Preprint] (2023). 10.48550/arXiv.2301.04104 (Accessed 6 October 2026). PMC12003158 40175544

[37] Y. Burda, H. Edwards, A. Storkey, O. Klimov, Exploration by random network distillation. arXiv [Preprint] (2018). 10.48550/arXiv.1810.12894 (Accessed 6 October 2026).

[38] L. E. Schulz, E. B. Bonawitz, Serious fun: Preschoolers play more when evidence is confounded. Dev. Psychol. **43**, 1045–1050 (2007). 10.1037/0012-1649.43.4.1045 17605535

[39] C. Cook, N. D. Goodman, L. E. Schulz, Where science starts: Spontaneous experiments in preschoolers’ exploratory play. Cognition **120**, 341–349 (2011). 10.1016/j.cognition.2011.03.003 21561605

[40] C. H. Legare, Exploring explanation: Explaining inconsistent evidence informs exploratory, hypothesis-testing behavior in young children. Child Dev. **83**, 173–185 (2012). 10.1111/j.1467-8624.2011.01691.x 22172010

[41] L. E. Schulz, The origins of inquiry: Inductive inference and exploration in early childhood. Trends Cogn. Sci. **16**, 382–389 (2012). 10.1016/j.tics.2012.06.004 22721579

[42] K. Nussenbaum, C. A. Hartley, Reinforcement learning across development: What insights can we draw from a decade of research? Dev. Cogn. Neurosci. **40**, 100733 (2019). 10.1016/j.dcn.2019.100733 PMC6974916 31770715

[43] F. Taffoni , Development of goal-directed action selection guided by intrinsic motivations: An experiment with children. Exp. Brain Res. **232**, 2167–2177 (2014). 10.1007/s00221-014-3907-z 24691755

[44] D. E. Berlyne, Novelty and curiosity as determinants of exploratory behaviour. Br. J. Psychol. **41**, 68 (1950).

[45] C. D. Smock, B. G. Holt, Children’s reactions to novelty: An experimental study of “curiosity motivation”. Child Dev. **33**, 631–642 (1962). 13914480

[46] F. Poli, M. Meyer, R. B. Mars, S. Hunnius, Contributions of expected learning progress and perceptual novelty to curiosity-driven exploration. Cognition **225**, 105119 (2022). 10.1016/j.cognition.2022.105119 PMC9194910 35421742

[47] E. G. Liquin, F. Callaway, T. Lombrozo, Developmental change in what elicits curiosity. Proc. Annu. Meet. Cogn. Sci. Soc. **43**, 1360–1366 (2021).

[48] A. Ruggeri, O. Stanciu, M. Pelz, A. Gopnik, E. Schulz, Preschoolers search longer when there is more information to be gained. Dev. Sci. **27**, e13411 (2024). 10.1111/desc.13411 37211720

[49] C. Addyman, D. Mareschal, Local redundancy governs infants’ spontaneous orienting to visual-temporal sequences. Child Dev. **84**, 1137–1144 (2013). 10.1111/cdev.12060 23432603

[50] F. Brändle, L. J. Stocks, J. B. Tenenbaum, S. J. Gershman, E. Schulz, Empowerment contributes to exploration behaviour in a creative video game. Nat. Hum. Behav. **7**, 1481–1489 (2023). 10.1038/s41562-023-01661-2 37488401

[51] D. E. Melnikoff, R. W. Carlson, P. E. Stillman, A computational theory of the subjective experience of flow. Nat. Commun. **13**, 2252 (2022). 10.1038/s41467-022-29742-2 PMC9042870 35474044

[52] E. Yiu, K. Allen, S. Ginosar, A. Gopnik, Empowerment gain and causal model construction: Children and adults are sensitive to controllability and variability in their causal interventions. Philos. Trans. R. Soc. A Math. Phys. Eng. Sci. **384**, 20250003 (2026). 10.1098/rsta.2025.0003 42130426

[53] S. J. Gershman, Deconstructing the human algorithms for exploration. Cognition **173**, 34–42 (2018). 10.1016/j.cognition.2017.12.014 PMC5801139 29289795

[54] A. F. Baranes, P. Y. Oudeyer, J. Gottlieb, The effects of task difficulty, novelty and the size of the search space on intrinsically motivated exploration. Front. Neurosci. **8**, 317 (2014). 10.3389/fnins.2014.00317 PMC4196545 25352771

[55] A. Ten, P. Kaushik, P. Y. Oudeyer, J. Gottlieb, Humans monitor learning progress in curiosity-driven exploration. Nat. Commun. **12**, 5972 (2021). 10.1038/s41467-021-26196-w PMC8514490 34645800

[56] E. Kosoy , Exploring exploration: Comparing children with RL agents in unified environments. arXiv [Preprint] (2020). 10.48550/arXiv.2005.02880 (Accessed 6 October 2026).

[57] R. Dubey, P. Agrawal, D. Pathak, T. L. Griffiths, A. A. Efros, Investigating human priors for playing video games. arXiv [Preprint] (2018). 10.48550/arXiv.1802.10217 (Accessed 6 October 2026).

[58] S. Bambach, D. Crandall, L. Smith, C. Yu, Toddler-inspired visual object learning. Adv. Neural Inf. Process. Syst. **31**, 1–10 (2018).

[59] E. Kosoy , “Learning causal overhypotheses through exploration in children and computational models” in Conference on Causal Learning and Reasoning, (PMLR, 2022), pp. 390–406.

[60] C. Salge, C. Glackin, D. Polani, Empowerment–An Introduction (Inception, Guided Self-Organization, 2014), pp. 67–114.

[61] F. Dupuis, W. Yu, F. M. Willems, “Blahut-Arimoto algorithms for computing channel capacity and rate-distortion with side information” in International Symposium on Information Theory, 2004. ISIT 2004. Proceedings, (IEEE, 2004), p. 179.

[62] S. Rathje , GPT is an effective tool for multilingual psychological text analysis. Proc. Natl. Acad. Sci. U.S.A. **121**, e2308950121 (2024). 10.1073/pnas.2308950121 PMC11348013 39133853

[63] Y. LeCun, A path towards autonomous machine intelligence version 0.9. 2, 2022–06-27. Open Review **62**, 1–62 (2022).

[64] H. Zhou, F. Dai, A. Gopnik, C. Wu, What is worth representing? Representational empowerment for continual model construction (Neurips, Main Track, Sydney, 2026). Workshop on Bridging Language, Agent, and World Models for Reasoning and Planning.

[65] H. Singh, N. Misra, V. Hnizdo, A. Fedorowicz, E. Demchuk, Nearest neighbor estimates of entropy. Am. J. Math. Manag. Sci. **23**, 301–321 (2003).

[66] M. Hessel , Rainbow: Combining improvements in deep reinforcement learning. Proc. AAAI Conf. Artif. Intell. **32**, 3215–3222 (2018).

[67] A. Lidayan , Humans in Crafter dataset. https://github.com/alyd/humans_in_crafter_data. Deposited 4 September 2026.

