# Supplementary material for: Intrinsically motivated humans and agents in open-world exploration
Source: Proc Natl Acad Sci U S A. 2026 Nov 4;123(45):e2612366123. doi: 10.1073/pnas.2612366123 (PMC13752353; doi:10.1073/pnas.2612366123)
Supplement: Supplementary file 1 [file pnas.2612366123.sapp.pdf]

Supporting Information for

Intrinsically Motivated Humans and Agents in Open-World Exploration

Aly Lidayan, Yuqing Du, Eliza Kosoy, Maria Rufova, Pieter Abbeel and Alison Gopnik

Aly Lidayan, Yuqing Du, Eliza Kosoy.
E-mail: dayan@berkeley.edu, yuqing_du@berkeley.edu, eko@berkeley.edu

This PDF file includes:

Supporting text
Figs. S1 to S3
Tables S1 to S2

Supporting Information Text

Utterance Classification. We used ChatGPT (`gpt-3.5-turbo`) to classify whether each private speech utterance expressed a goal and/or a question, prompting for reasoning before each classification. The prompts are given below, and sample responses are shown in Tables S1 and S2.

Goal Prompt

```
The following text is a part of a transcript from a person playing a game for the first time. Is the person
     expressing a goal? Provide reasoning and answer with Finish[1] for yes or Finish[0] for no or if it cannot
     be determined.

EX: I need more woooOODD! A: This is a goal because the person is expressing their need for wood resources.
     Finish[1]

EX: Wait can I even get, oh yea. A: This is not a goal because the person is stating an incomplete thought.
     Finish[0]

EX:
```

Question Prompt

```
The following text is a part of a transcript from a person playing a game for the first time. Is the person
     asking a question? Provide reasoning and answer with Finish[1] for yes or Finish[0] for no or if it cannot
     be determined.

EX: why is the blue button not working? A: This is a question because the person is asking a question about the
      game. Finish[1]

EX: Yes. A: This is not a question because it is just a yes or no answer. Finish[0]

EX:
```

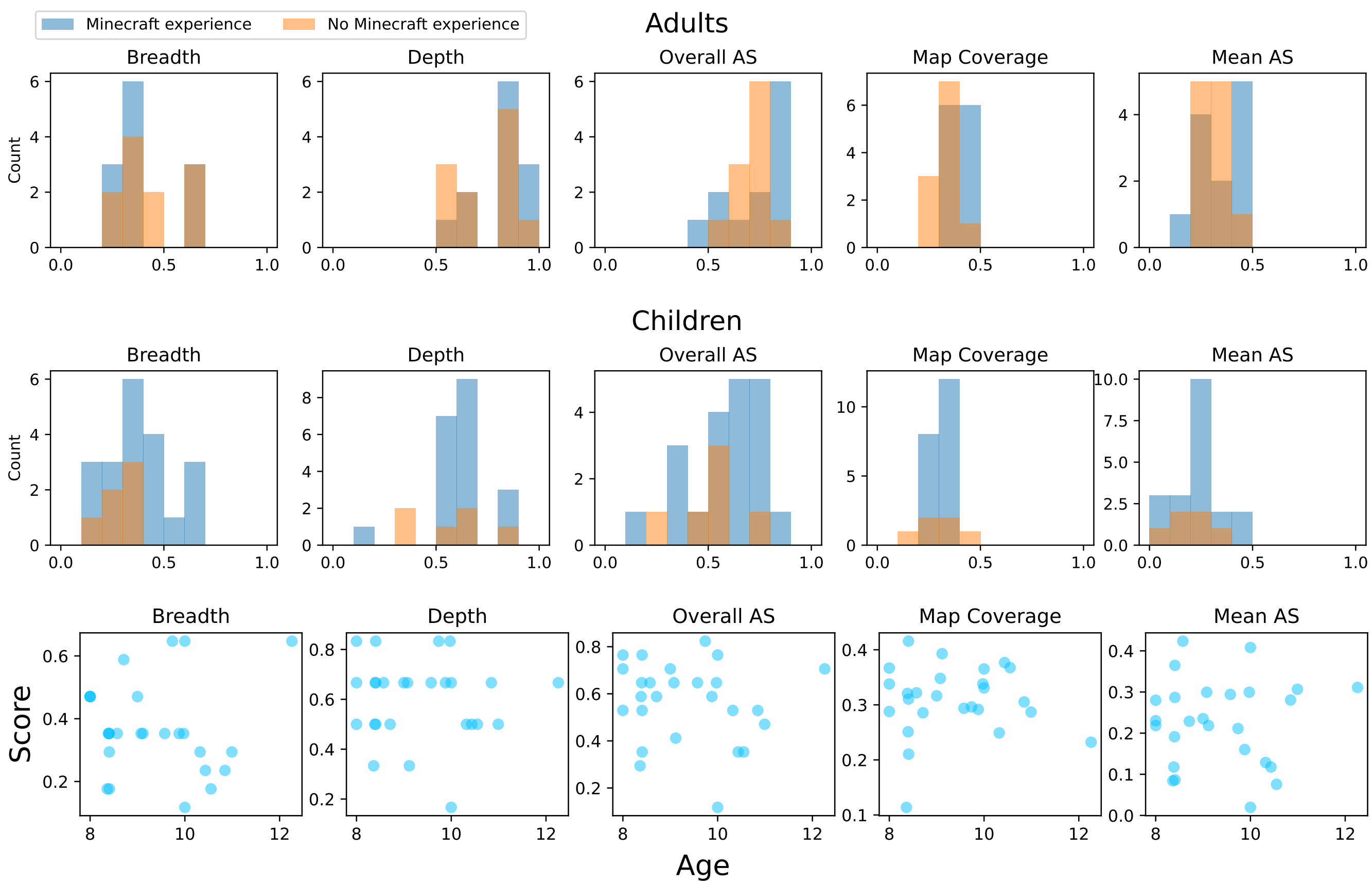

Fig. S1. Crafter exploration scores of the human participants plotted by prior Minecraft experience (top two rows) and the age of the children (bottom). No significant differences or correlations were found at $p<0.05$ by Welch's t-test and Pearson's correlation analysis, except for a small difference for adults' Map Coverage score.

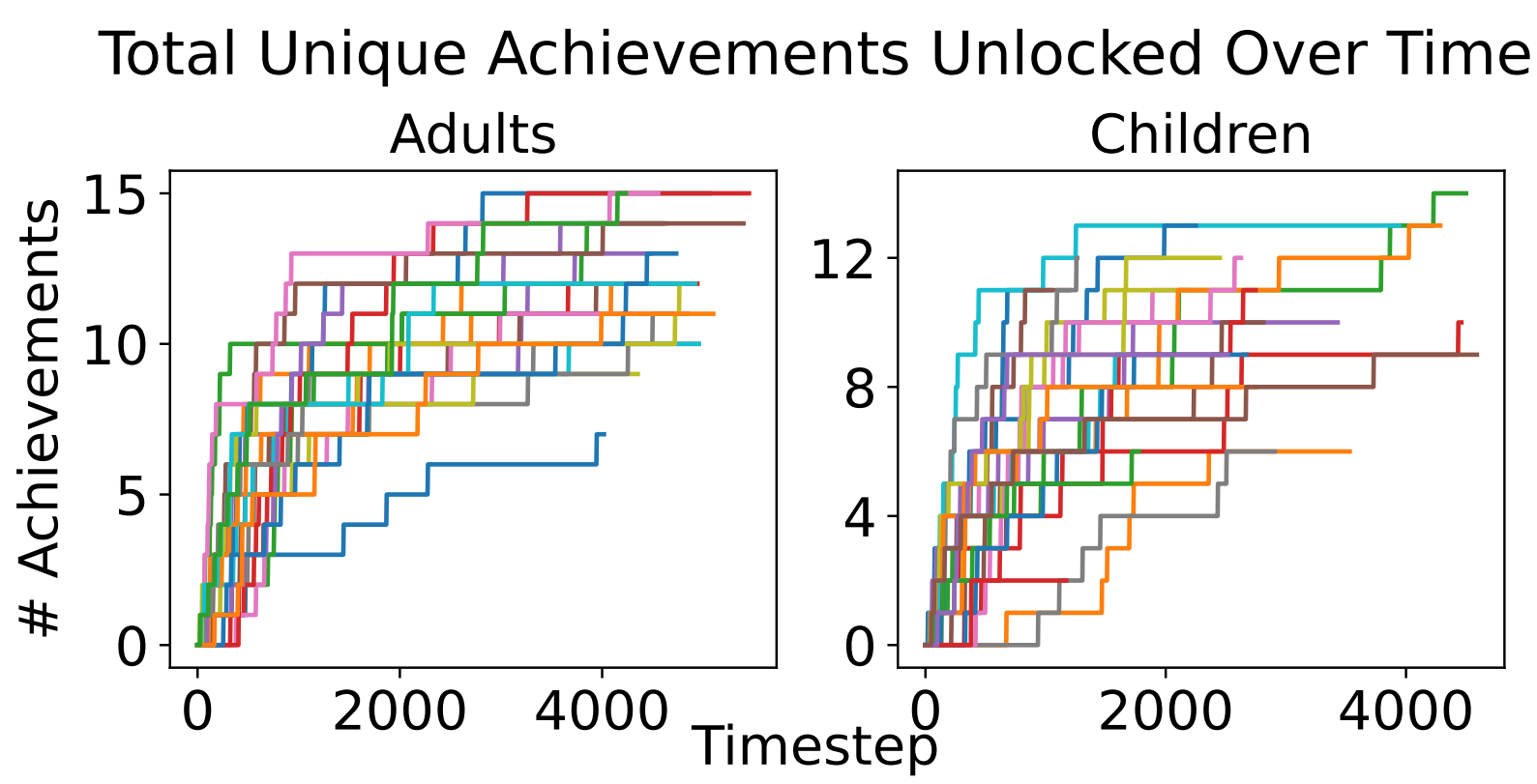

Fig. S2. The number of unique achievements reached over time for a random subset of the human participants, concatenating all episodes of each participant's gameplay in chronological order. Each timestep in the game lasts about 0.2 seconds. Each participant's curve ends when they voluntarily stop playing–children often quit sooner than the adults. Plotting each participant separately shows the wide individual variation in exploration trajectories.

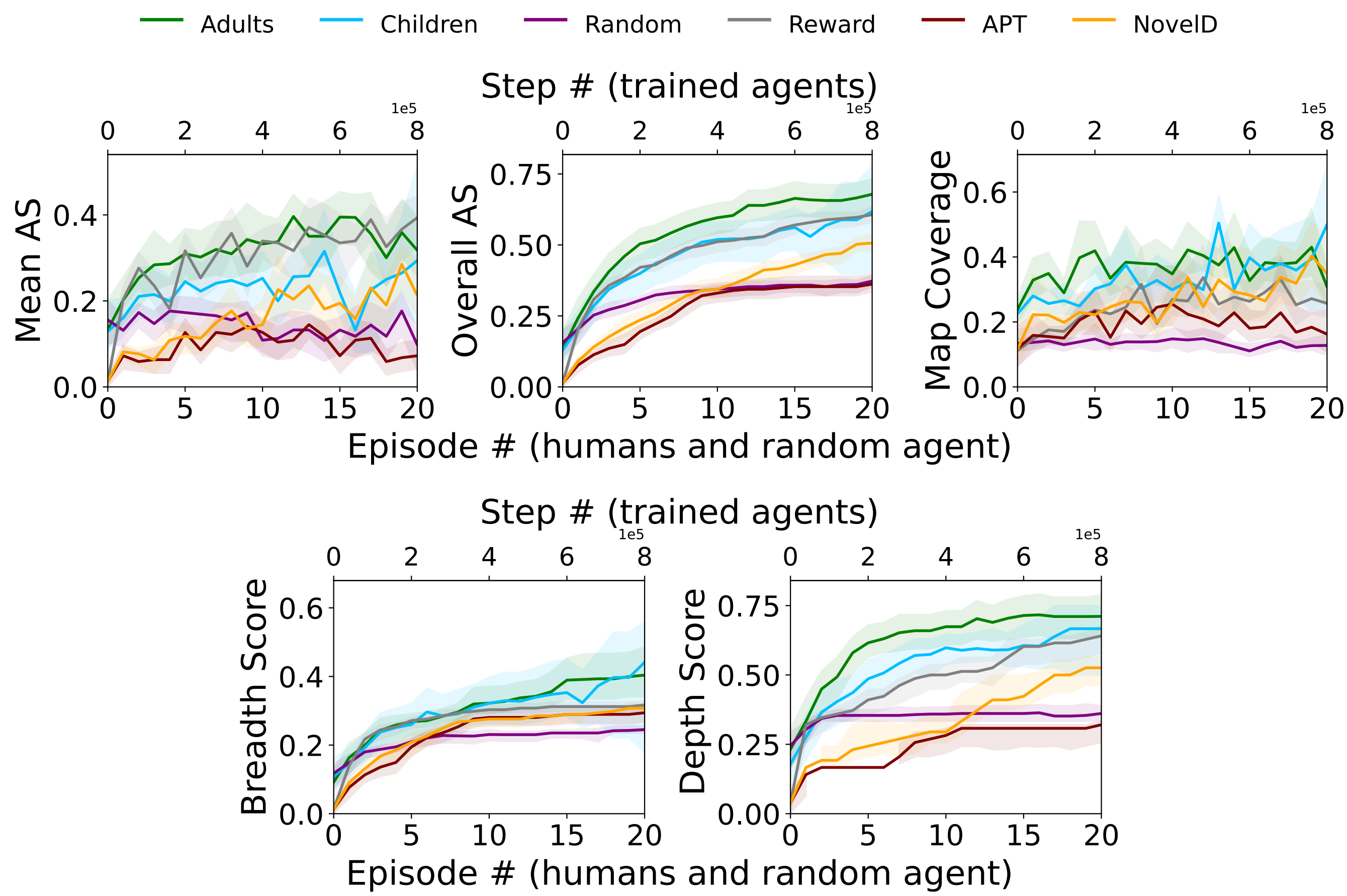

Fig. S3. The progression of exploration scores over time for each group of humans and AI agents. Shaded regions indicate bootstrapped 95% CIs. Agents are much less sample-efficient than humans, reaching similar performance only after more than 100× as many environment interactions.

Table S1. Sample goal classification responses.

Input utterance	Response
sooo, next thing build the table with your wood. also make the pickaxe.	This is a goal because the person is outlining specific tasks they want to accomplish in the game. Finish[1]
i should run, oh no, oh noooo.	This is not a clear goal as the person is expressing panic rather than a specific objective. Finish[0]
um maybe i should just figure out what happens when i die	This is a goal because the person is expressing a desire to figure out what happens when they die in the game. Finish[1]
which button to go left? red.	This is not a goal because the person is asking a question about game controls. Finish[0]

Table S2. Sample question classification responses.

Input utterance	Response
how much stone do i need for stone pickaxe again?	This is a question because the person is asking for information about the game. Finish[1]
it's hard, it's hard. what? ready. it's not moving.	This is not a clear question as the person seems to be expressing frustration and confusion rather than asking a direct question. Finish[0]
wait, can i hit this thing?	This is a question because the person is asking if they can interact with something in the game. Finish[1]
i need to drink water. drink drink.	This is not a question, it is a statement. Finish[0]
where is stone? great.	This is a question because the person is asking where something is in the game. Finish[1]
